# Comparative Analysis of Corneal Densitometry Changes Following Standard Versus Accelerated Corneal Cross-Linking Protocol

**DOI:** 10.3390/medicina61111928

**Published:** 2025-10-28

**Authors:** Lorena Karla Šklebar, Sonja Jandroković, Miro Kalauz, Ivan Škegro, Dina Lešin Gaćina, Iva Bešlić, Ivana Gabrić, Niko Tomljanović, Božana Mrvelj, Sania Vidas Pauk

**Affiliations:** 1 Department of Ophthalmology, University Hospital Centre Zagreb, 10000 Zagreb, Croatia; lkskleba@kbc-zagreb.hr (L.K.Š.); sonja.jandrokovic@kbc-zagreb.hr (S.J.); miro.kalauz@gmail.com (M.K.); ivanskegro@yahoo.com (I.Š.); dina.lesin@kbc-zagreb.hr (D.L.G.); iva.beslic@kbc-zagreb.hr (I.B.); ivanajonjic91@gmail.com (I.G.); mrveljbozana@gmail.com (B.M.); 2 School of Medicine, University of Zagreb, 10000 Zagreb, Croatia; 3 Ghetaldus Eye Clinic, 10000 Zagreb, Croatia; nikotomljanovic96@gmail.com

**Keywords:** keratoconus, corneal cross-linking, corneal densitometry, corneal haze, Scheimpflug imaging

## Abstract

*Background and Objectives*: The aim of this study is to evaluate and compare changes in corneal densitometry after standard (30 min exposure time of 3 mW/cm^2^ UVA) and accelerated (10 min exposure time of 9 mW/cm^2^ UVA) protocols of corneal cross-linking (CXL) in patients with progressive keratoconus. *Materials and Methods*: This study included a total of 38 eyes of 38 patients divided into two equal-sized subgroups. CXL was performed in one group according to the standard epithelium-off protocol (30 min, 3 mW/cm^2^ UVA) and in the other group according to an accelerated epithelium-off protocol (10 min, 9 mW/cm^2^ UVA). Scheimpflug imaging was used to evaluate corneal densitometry in the anterior, central, and posterior corneal layers in three concentric zones (0–2 mm, 2–6 mm, and 6–10 mm) at baseline and 1, 3, and 9 months after surgery. *Results:* This study included 38 patients divided into two subgroups of 19. One group of patients underwent standard and the other accelerated CXL protocol. Participants in the accelerated group were significantly older (*p* < 0.001). 9 months after CXL treatment, the accelerated group showed higher central and posterior corneal densitometry values, but, after adjusting for age and baseline values, ANCOVA analysis revealed no significant intergroup differences. Both protocols led to overall reductions in corneal densitometry over time. *Conclusions*: Both the standard and accelerated CXL protocols induce transient corneal haze, which can be objectified by increased corneal densitometry values in first three months post-CXL. The dynamics of the onset and recovery of postoperative corneal haze are comparable and similar in both protocols.

## 1. Introduction

Keratoconus is an ectatic corneal disease which most often affects younger individuals and has variable prevalence, occurring equally in both men and women [1]. It leads to progressive corneal thinning, irregular astigmatism, and decreased visual acuity, which can significantly reduce the patient’s quality of life [2]. Although mild-to-moderate cases can be treated with contact lenses, cases of progressive keratoconus should be managed surgically to prevent more severe forms [3]. The most common type of surgical treatment of keratoconus is corneal cross-linking (CXL), which effectively slows or halts progression of corneal thinning. This method utilizes riboflavin and ultraviolet A (UVA) light to induce cross-linking of the corneal collagen fibres, resulting in increased corneal rigidity and stiffness [4]. The original protocol, also called the standard, conventional, or “Dresden” protocol, involves corneal epithelial debridement, after which the corneal stroma is saturated with riboflavin, followed by 30 min of 3 mW/cm^2^ intensity UVA illumination [4,5]. However, although effective, this protocol has its disadvantages, such as long procedure time and pain or discomfort due to corneal epithelial debridement [5,6,7]. Therefore, alternative protocols have been introduced, aiming to achieve comparable biomechanical and clinical outcomes in a shorter time. Based on the Bunsen–Roscoe law of reciprocity, accelerated protocols were developed [8,9,10]. Due to higher UVA illumination intensity, these protocols managed to achieve similar results to the “Dresden” protocol, but with a shorter duration of UVA illumination [11,12]. In many studies, an accelerated method which uses a 10 min corneal exposure time of 9 mW/cm^2^ UVA intensity after initial corneal debridement shows comparable clinical outcomes in halting the progression of keratoconus and stiffening the cornea compared to the “Dresden” protocol [4,13,14,15,16,17,18,19]. Regardless of the protocol, transient loss of corneal transparency, also known as corneal haze, is a common and expected finding after CXL. Corneal haze occurs as a result of microstructural changes in the cornea, such as keratocyte loss and remodelling of collagen lamellae, and its resolution is of paramount importance in recovering visual function after CXL [20,21]. Even a subtle corneal haze which cannot be detected by slit lamp can have a great impact on visual quality by reducing contrast sensitivity or inducing glare and higher-order aberrations [22], consequently creating functional consequences.

Corneal transparency or haze is objectified and quantified by corneal densitometry. Corneal densitometry mapping is based on the amount of backscattered light in different regions of the cornea measured by non-invasive Scheimpflug analysis with the Oculus Pentacam device [23]. Most studies in this field are coherent and report that CXL most commonly induces a transient increase in haze or densitometry values, typically peaking within the first few postoperative months and gradually declining over time [22,24,25,26,27,28,29]. After the CXL procedure, the process of redistribution of corneal collagen cross-links differs between protocols, therefore potentially causing different light-scattering patterns of the cornea, resulting in alteration of densitometry values.

Some studies which have evaluated differences in corneal densitometry between different accelerated protocols suggest subtle variations in haze dynamics depending on the irradiation parameters and epithelial debridement [28,30,31,32]. However, although there are some studies directly comparing clinically significant corneal haze in an accelerated protocol (10 min, 9 mW/cm^2^ UVA) and the Dresden protocol, the literature is lacking in studies comparing the two protocols with respect to corneal densitometry, which is an objective measurement of corneal transparency. Available studies which have directly compared corneal haze between the standard “Dresden” and accelerated CXL protocols present findings of slightly higher incidence of haze after the “Dresden” protocol compared to the accelerated protocol (10 min, 9 mW/cm^2^ UVA), while changes in corneal densitometry were mostly localized in the central concentric zones (0–6 mm) [31,33,34] in the early postoperative period. This underscores the need for individualization of treatment choice, taking into consideration not only long-term but also short-term visual demands of our patients.

Overall, although corneal densitometry is increasingly recognized as an important outcome measure, evidence comparing its evolution and resolution after different CXL protocols remains limited. Therefore, the aim of this study is to evaluate and compare changes in corneal densitometry after standard (30 min exposure time of 3 mW/cm^2^ UVA) and accelerated (10 min exposure time of 9 mW/cm^2^ UVA) protocols in patients with keratoconus.

## 2. Materials and Methods

### 2.1. Ethics

This prospective, longitudinal study was performed in the Department of Ophthalmology, University Clinical Hospital Centre Zagreb, following the Declaration of Helsinki and was approved by the Hospital’s Ethics Committee (protocol code 8.1-25/156-2 number 02/013 AG). The patients received written and oral information about the intervention and study and signed written informed consent.

### 2.2. Subjects and Methods

A sample size calculation indicated that to detect a medium effect size (f = 0.25) for differences in continuous variables between two independent groups across four measurement points, with an alpha level of 0.05 and a statistical power of 0.95, a minimum of 36 participants (18 per group) were required (G*Power, version 3.1.9.7).

This study enrolled 38 eyes of 38 patients (one eye per patient), divided into two groups of 19 each, assigning every second eligible patient alternately to one of the two treatment groups. We included patients with a documented diagnosis of progressive keratoconus, with a minimum corneal thickness of 400 μm without the corneal epithelium as determined by Scheimpflug tomography (Pentacam device, Oculus Optikgeräte GmbH, Wetzlar, Germany). Non-inclusion criteria included central corneal scarring, history of chemical injury, severe corneal infection, ocular surface disease, pregnancy, or lactation during the study period.

Diagnosis and confirmation of keratoconus progression were performed by a corneal specialist, the last author. Progression of keratoconus was defined according to the Global Consensus on Keratoconus and Ectatic Diseases [35]. In line with these criteria, “ectasia progression” required reproducible changes in at least two of the following parameters, which included steepening of the anterior corneal surface, steepening of the posterior corneal surface, and thinning and/or an increased rate of pachymetry progression from the periphery to the thinnest point, with magnitudes exceeding the inherent variability of the measurement system. These changes had to be consistent across series of measurements to be considered indicative of progression.

Once progression was confirmed, patients were allocated to undergo either a conventional epithelium-off CXL protocol (30 min of UVA exposure at 3 mW/cm^2^) or an accelerated protocol (10 min of UVA exposure at 9 mW/cm^2^).

Prior to the procedure, we administered anaesthesia topical 1% tetracaine, after which 1% pilocarpine was instilled to induce pharmacological miosis. Then, under sterile conditions, using a blunt hockey knife, the corneal epithelium was mechanically debrided. The exposed stroma was then saturated with riboflavin 0.1% combined with hydroxypropyl methylcellulose 1.1% (MedioCROSS M, MedioCROSS, Medio-Haus-Medizinprodukte GmbH, Kiel, Germany). We applied this solution several times over a period of 10 min at 2 min intervals to ensure adequate absorption. After that, UVA irradiation was performed according to the chosen treatment protocol. The accelerated protocol group was exposed to UVA radiation of 9 mW/cm^2^ for 10 min, while the standard Dresden protocol group was exposed to UVA radiation of 3 mW/cm^2^ continuously for 30 min. In order to maintain corneal saturation during UVA irradiation, additional drops of riboflavin and methylcellulose solution were added every 5 min. After treatment, the cornea was rinsed thoroughly with balanced salt solution. Topical dexamethasone and a broad-spectrum double-antibiotic (neomycin sulfate, and polymyxin B sulfate) were then instilled, and a therapeutic soft bandage contact lens was placed, which remained in situ for six days.

Patients were prescribed preservative-free topical antibiotics in the immediate postoperative period (moxifloxacin drops) for up to six days. After removal of the therapeutic bandage soft contact lens, topical antibiotics were continued. Once this regimen was completed, patients were prescribed preservative-free dexamethasone drops alone, administered once or twice daily for one month.

Baseline assessment was performed on the day of the procedure, followed by postoperative evaluations at 1 month, 3 months, and 9 months. At each visit, patients underwent a complete ophthalmic examination including slit-lamp biomicroscopy and corneal densitometry (Pentacam device, Oculus Optikgeräte GmbH, Wetzlar, Germany). Corneal densitometry data, expressed in greyscale units (GSU), was analysed in all three layers of the cornea (anterior 120 μm, central and posterior 60 μm) in three central zones of the cornea (0–2 mm, 2–6 mm, and 6–10 mm). All densitometric measurements were performed by the last author and subsequently reviewed by the first author. To avoid contact-lens-related bias, patients were instructed to discontinue lens wear for at least 5–7 days prior to each scheduled visit.

### 2.3. Statistical Analyses

Categorical data were presented as absolute and relative frequencies, and their differences were tested using Fisher’s exact test. The normality of distribution of continuous variables was assessed with the Shapiro–Wilk test. Because most variables did not follow a normal distribution and the sample size was relatively small, the results were summarized using the median and interquartile range (IQR). Accordingly, comparisons between groups were performed using non-parametric tests, including the Mann–Whitney U test (with the difference and 95% CI reported) for independent samples and Friedman’s test for repeated measures (with Conover’s post hoc test). To control for multiple comparisons, *p*-values were adjusted using the Benjamini–Hochberg false discovery rate (FDR) correction. In addition, to account for potential confounding by age and baseline densitometry, a multivariate analysis of covariance (ANCOVA) was performed for each densitometry parameter at 1, 3, and 9 months after CXL, with age and baseline values entered as covariates [36]. All *p*-values were two-tailed. The level of significance was set at alpha = 0.05. Statistical analyses were performed using MedCalc^®^ Statistical Software version 23.3.7 (MedCalc Software Ltd., Ostend, Belgium; https://www.medcalc.org; 2025). The study report was prepared in accordance with the guidelines for reporting research results in biomedicine and health sciences [37].

## 3. Results

This study included 38 patients divided into two groups of 19 patients depending on the protocol used for CXL. No significant differences were observed between groups in terms of sex or operated eye according to the treatment protocol (Table 1). Participants assigned to the accelerated protocol were significantly older than those assigned to the standard protocol (Mann–Whitney U test, *p* < 0.001) (Table 2).

Prior to treatment, participants in the accelerated protocol group demonstrated greater corneal densitometry values across several parameters compared with the standard protocol group. However, after correction for multiple comparisons using the Benjamini–Hochberg false discovery rate (FDR) method, only posterior corneal densitometry at 6–10 mm remained statistically significant (Mann–Whitney U test, *p* = 0.04) (Supplementary Material, Table S1).

At one and three months following CXL, there were no significant differences in corneal densitometry between the standard and accelerated protocol groups (Supplementary Material, Tables S2 and S3).

At nine months post-CXL, participants in the accelerated protocol group had significantly higher values of central corneal densitometry at 6–10 mm (Mann–Whitney U test, *p* = 0.02), posterior corneal densitometry (Mann–Whitney U test, *p* = 0.006), and total corneal densitometry at 6–10 mm (Mann–Whitney U test, *p* = 0.02) compared with those in the standard protocol group (Supplementary Material, Table S4).

Within the standard protocol group, significant changes were observed in all corneal densitometry values across time points.

Within the accelerated protocol group, significant changes were also observed in all corneal densitometry values across time points, except for posterior corneal densitometry at 6–10 mm (Supplementary Material, Table S5).

The results of additional analyses using analysis of covariance (ANCOVA) with age and baseline densitometry values as covariates for all densitometry parameters at 1, 3, and 9 months after CXL (presented in Supplementary Material, Table S6) showed that after adjustment for age and baseline densitometry values, differences between the standard (3 mW) and accelerated (9 mW) CXL protocols remained non-significant. Age and baseline values were significant covariates mainly at one month. Baseline densitometry was consistently significant in the 6–10 mm zones, and age was significant at one month for anterior densitometry values.

In both treatment protocols, corneal densitometry values showed a general decrease from baseline to nine months after CXL (Supplementary Material, Table S7). In the standard protocol group, significant reductions were observed in the anterior (0–2 mm and 6–10 mm), central (0–2 mm and 2–6 mm), and total (0–2 mm) corneal layers. In the accelerated protocol group, significant decreases were found only in the outer 6–10 mm zone of the anterior, central, and total corneal layers. No other within-group differences remained significant after FDR correction.

## 4. Discussion

This study aimed to compare corneal densitometry values of patients with keratoconus who underwent corneal cross-linking following either the standard (3 mW/cm^2^ for 30 min) or an accelerated (9 mW/cm^2^ for 10 min) protocol.

Our findings demonstrated that, as expected, densitometry values after CXL increased in both treatment groups, peaking in the period of one to three months postoperatively, and decreasing in the period of nine months postoperatively (Supplementary Material, Table S5). At baseline, patients who had received the accelerated treatment had a greater value of densitometry in all three layers (anterior, central, and posterior) of the cornea in the 6–10 mm zone and in the posterior layer in the 0–2 mm zone compared to those treated with the standard protocol (Supplementary Material, Table S1). This could be the reason for significant intergroup differences at the nine-month follow-up point (Supplementary Material, Table S4), considering that statistically significant differences have occurred in the same zones and layers as in the preoperative measurements (except the posterior layer in the 0–2 mm zone), which is in accordance with the expected finding of gradual return to baseline values. Interestingly, at the one- and three-month follow-up points (Supplementary Material, Tables S2 and S3), there was not a statistically significant difference in densitometry values of any layer or zones of the cornea. The differences in baseline values could be the result of a significant age difference between two groups, with a median age of 29 in the accelerated group compared to a median age of 23 years in the standard group. This assumption is further confirmed by findings of Dhubhghaill et al., who have stated that with age, the most significant increase in densitometry is in the 6–10 mm zone, which likely represents the development of age-related corneal limbal degenerations [23]. Regarding general course of corneal transparency recovery after CXL, our findings are in accordance with the previous literature, demonstrating that in both protocols there is a transient post-CXL haze with increase in corneal densitometry in all corneal layers and zones, as a result of corneal remodelling. However, when comparing postoperative corneal transparency between the standard and accelerated protocol, our results suggest that corneal densitometry values followed a similar postoperative course between the two protocols, with initial elevation in corneal densitometry values at one to three months and subsequent decline toward baseline by nine months.

These findings differ from previous research which found that the standard protocol caused a slightly higher incidence of postoperative haze compared to the accelerated protocol [31,33,34]. In our study, we did not observe such variations, and the transparency recovery was similar after both treatments. Furthermore, in the 9-month postoperative period, we observed significantly higher values of densitometry in the accelerated group; however, these results were explained by higher baseline values of the same group, as was previously mentioned.

Although Kortuem et al. did not present densitometry data in their paper, they observed that patients who were treated with the standard protocol had greater postoperative corneal opacity in the period from three to six months postoperatively [33]. Furthermore, when comparing densitometric changes following the standard Dresden vs. accelerated (10 min, 9 mW/cm^2^) corneal UVA cross-linking protocols, Prinz et al. showed that the standard protocol led to an increase in densitometric values of the anterior central corneal zone over a period of 24 months [34]. Our results have not shown such differences and indicate that the dynamics of postoperative corneal opacification is similar when applying both the standard Dresden and the accelerated (10 min, 9 mW/cm^2^) protocol, with densitometry values increasing in the first few months and then returning to baseline values. These differences may be explained by variations in follow-up period, characteristics of the study groups, mainly in age, and study design (retrospective vs. prospective). However, our results could have significant clinical implications for the management of patients with keratoconus. Clarifying how both protocols have a similar effect on corneal clarity and, therefore, potentially on visual acuity, physicians can individualize their protocol choice based on each patient’s characteristics, such as corneal thickness, age, compliance, or other clinical factors.

This research has several good features. The study design is prospective, with pre-defined protocols and standardized imaging, treatment, and follow-up of patients, which allows the study to be reproduced and objectified. However, we must also address the limitations of this study. Although the sample size is sufficient according to the power analysis calculated for a medium effect size (f = 0.25), it is still relatively small, and subgroup analysis is limited. The significant difference in average age between the groups is a potential confounder of this study, given that older patients generally have higher corneal densitometry values [23]. Although ANCOVA was performed to address this confounder, this should be mentioned as a limitation. Although the follow-up period of 9 months is long enough to analyse the most significant changes in corneal clarity and to clearly compare the densitometry trend after both protocols, it is not long enough to determine long-term effects. A longer follow-up period would enable the detection of possible differences on corneal clarity between the protocols. Also, the study was performed in a single centre and was not fully randomized using a randomization protocol; instead, every second eligible patient was assigned alternately to one of the two treatment groups, which could lead to potential bias. Another limitation is lack of correlation of corneal densitometry data to functional outcomes like best-corrected visual acuity, contrast sensitivity, higher-order aberrations, or patient-reported symptoms like glare. Future research in this area should address these limitations. Larger, randomized clinical trials with a significantly longer follow-up period and with comparative analysis of other clinical parameters could determine whether there is any difference in the onset, progression, and resolution of corneal opacities after CXL with an even higher level of certainty.

## 5. Conclusions

In conclusion, both the standard Dresden and accelerated (10 min, 9 mW/cm^2^) corneal cross-linking protocols induce transient corneal haze, which can be objectified by increased corneal densitometry values. The dynamics of the onset and recovery of postoperative corneal haze are comparable and similar in both protocols. Such findings suggest that both protocols are safe for use even in patients with high visual demands. These results also allow the physicians to make the choice of protocol based primarily on the individual characteristics of each patient, such as age, corneal thickness, and ability to cooperate, without concerns about postoperative corneal clarity.

## Figures and Tables

**Table 1:** Baseline characteristics of participants by treatment protocol.

	Number (%) of Participants			*p* *
	Standard Protocol	Accelerated Protocol	Total	
**Gender**				
Men	12 (63.2)	17 (89.5)	29 (76.3)	0.12
Women	7 (36.8)	2 (10.5)	9 (23.7)	
Eye				
Right	9 (47.4)	11 (57.9)	20 (52.6)	0.52
Left	10 (52.6)	8 (42.1)	18 (47.4)	

* Fisher’s exact test.

**Table 2:** Differences in age between participant groups.

	Median (Interquartile Range)		^†^ Difference	95% Confidence Interval	p *
	Standard Protocol	Accelerated Protocol			
Age (years)	23 (19–26)	29 (27–38)	7	3 to 12	<0.001

* Mann–Whitney U test; ^†^ Hodges–Lehmann median difference.

## Data Availability

The original contributions presented in this study are included in the article. Further inquiries can be directed to the corresponding author.

## Supplementary Materials

The following supporting information can be downloaded at: https://www.mdpi.com/article/10.3390/medicina61111928/s1, Table S1. Pre-treatment differences in corneal densitometry between the standard and accelerated CXL protocols.; Table S2. Differences in corneal densitometry between the standard and accelerated CXL protocols at one-month post-procedure.; Table S3. Differences in corneal densitometry between the standard and accelerated CXL protocols at three months post-procedure.; Table S4. Differences in corneal densitometry between the standard and accelerated CXL protocols at nine months post-procedure.; Table S5. Corneal densitometry changes across time points for the standard and accelerated CXL protocols.; Table S6. ANCOVA results for corneal densitometry after CXL, adjusted for age and baseline densitometry.; Table S7. Within-group changes in corneal densitometry before and nine months after CXL for the standard and accelerated protocols.

## Abbreviations

The following abbreviations are used in this manuscript:

ANCOVA	Analysis of Covariance
CI	Confidence Interval
CXL	Corneal Cross-Linking
GSU	Greyscale Units
HPMC	Hydroxypropyl Methylcellulose
UVA	Ultraviolet A
