# Supplementary material for: Comparative Analysis of Corneal Densitometry Changes Following Standard Versus Accelerated Corneal Cross-Linking Protocol
Source: Medicina (Kaunas). 2025 Oct 28;61(11):1928. doi: 10.3390/medicina61111928 (PMC12653996; doi:10.3390/medicina61111928)
Supplement: Supplementary file 1 [file medicina-61-01928-s001.zip › medicina-3931727-supplementary.pdf]

Table S1. Pre-treatment differences in corneal densitometry between the standard and accelerated CXL protocols.

Before CXL	Median (interquartile range)		†Difference	95% CI	*P**
	Standard protocol	Accelerated protocol			
Anterior (120 µm) cornea 0 – 2 mm	21.3 (19.5 – 23.2)	21.1 (20.1 – 23.4)	0.2	-1.5 to 2.2	0.94
Anterior (120 µm) cornea 2 – 6 mm	17.8 (17.3 – 18.7)	18.3 (17.8 – 19.7)	0.5	-0.4 to 1.6	0.48
Anterior (120 µm) cornea 6 – 10 mm	15.8 (14.4 – 17.3)	17.4 (16.3 – 18.3)	1.3	0 to 2.3	0.10
Center cornea layer 0 – 2 mm	15.5 (15.4 – 16.8)	16.2 (15.6 – 17.1)	0.3	-0.6 to 1.1	0.62
Center cornea layer 2 – 6 mm	13.8 (13.3 – 14.7)	14.2 (13.6 – 14.9)	0.4	-0.4 to 1.1	0.98
Center cornea layer 6 – 10 mm	12.4 (11.9 – 13.7)	14.0 (13.2 – 15.3)	1.4	0.3 to 2.4	0.06
Posterior (60 µm) cornea 0 – 2 mm	12.3 (11.8 – 13.3)	12.6 (11.9 -13.3)	0.3	-0.5 to 0.9	0.98
Posterior (60 µm) cornea 2 – 6 mm	11.4 (11.1 – 12.1)	11.9 (11.5 – 12.5)	0.4	-0.2 to 0.9	0.98
Posterior (60 µm) cornea 6 – 10 mm	11.1 (10.6 – 12.5)	12.8 (12.3 – 13.7)	1.6	0.3 to 2.4	**0.04**
Total cornea densitometry 0 – 2 mm	16.3 (15.7 – 17.2)	16.6 (16.1 – 17.9)	0.4	-0.5 to 1.4	0.98
Total cornea densitometry 2 – 6 mm	14.3 (13.8 – 15.3)	15.0 (14.5 – 15.5)	0.5	-0.3 to 1.2	0.31
Total cornea densitometry 6 – 10 mm	13.2 (12.7 – 14.5)	14.7 (14.1 – 15.9)	1.4	0.3 to 2.3	0.06

CI – Confidence interval; *Mann–Whitney U test; †Hodges–Lehmann median difference. P values were adjusted using the Benjamini–Hochberg false discovery rate (FDR) correction. Bold denotes statistical significance after adjustment.

Table S2. Differences in corneal densitometry between the standard and accelerated CXL protocols at one-month post-procedure.

1 month after CXL	Median (interquartile range)		†Difference	95% CI	*P**
	Standard protocol	Accelerated protocol			
**Anterior (120 µm) cornea 0 – 2 mm**	**30.0 (25.7 – 31.2)**	**27.5 (25.5 – 29.9)**	-1.4	-4.1 to 1.8	0.78
Anterior (120 µm) cornea 2 – 6 mm	22.9 (22.2 – 26.1)	23.5 (21.6 – 25.4)	-0.2	-2.6 to 2.2	0.98
Anterior (120 µm) cornea 6 – 10 mm	17.5 (16.6 – 18.8)	18.3 (16.6 – 19.6)	0.7	-1.7 to 2.1	0.98
Center cornea layer 0 – 2 mm	23.1 (22.0 – 23.8)	22.9 (19.5 – 28.1)	0.5	-2.6 to 4.4	0.98
Center cornea layer 2 – 6 mm	19.7 (18.4 – 22.9)	19.0 (18.2 – 20.9)	-1	-3.2 to 1.1	0.98
Center cornea layer 6 – 10 mm	14.1 (12.9 – 14.6)	15.1 (12.9 – 16.1)	0.5	-1 to 2	0.98
Posterior (60 µm) cornea 0 – 2 mm	17.2 (14.9 – 19.6)	18.2 (14.7 – 19.5)	0.1	-2.8 to 2.5	0.98
Posterior (60 µm) cornea 2 – 6 mm	15.7 (14.5 – 17.4)	14.8 (13.6 – 15.6)	-1.1	-2.6 to 0.5	0.98
Posterior (60 µm) cornea 6 – 10 mm	12.1 (10.8 – 13.1)	13.4 (11.5 – 14.8)	0.9	-0.3 to 2.4	0.98
Total cornea densitometry 0 – 2 mm	22.3 (22.1 – 25.0)	22.5 (20.1 – 25.8)	-0.3	-3.4 to 2.5	0.98
Total cornea densitometry 2 – 6 mm	19.4 (18.7 – 22.1)	18.9 (18.1 – 20.9)	-0.6	-2.4 to 1.2	0.98
Total cornea densitometry 6 – 10 mm	14.8 (13.6 – 15.3)	15.8 (13.7 – 16.8)	0.8	-1 to 2	0.84

CI – Confidence interval; *Mann–Whitney U test; †Hodges–Lehmann median difference. P values were adjusted using the Benjamini–Hochberg false discovery rate (FDR) correction.

Table S3. Differences in corneal densitometry between the standard and accelerated CXL protocols at three months post-procedure.

3 months after CXL	Median (interquartile range)		†Difference	95% CI	*P**
	Standard protocol	Accelerated protocol			
Anterior (120 µm) cornea 0 – 2 mm	29.8 (25.1 – 35.2)	28.4 (25.7 – 29.9)	-1.7	-6.2 to 2	0.77
Anterior (120 µm) cornea 2 – 6 mm	24.5 (21.0 – 27.4)	22.9 (20.7 – 24.3)	-1.8	-3.9 to 0.8	0.52
Anterior (120 µm) cornea 6 – 10 mm	17.1 (15.9 – 18.5)	17.3 (16.3 – 18.1)	0.1	-1.4 to 1.3	0.98
Center cornea layer 0 – 2 mm	24.3 (19.3 – 25.4)	21.8 (19.7 – 24.5)	-1	-3.3 to 1.6	0.86

Center cornea layer 2 – 6 mm	18.7 (16.8 – 19.8)	17.4 (15.6 – 18.5)	-1.2	-2.6 to 0.2	0.42
Center cornea layer 6 – 10 mm	12.8 (12.6 – 14.5)	13.8 (13.0 – 15.0)	0.5	-0.4 to 1.6	0.98
Posterior (60 µm) cornea 0 – 2 mm	16.7 (14.6 – 18.0)	15.3 (14.1 – 18.3)	-0.5	-2.4 to 1.3	0.86
Posterior (60 µm) cornea 2 – 6 mm	13.8 (12.9 – 14.6)	13.1 (12.2 – 14.2)	-0.6	-1.5 to 0.4	0.66
Posterior (60 µm) cornea 6 – 10 mm	11.5 (10.9 – 13.1)	12.9 (11.9 – 14.0)	0.9	-0.2 to 2	0.98
Total cornea densitometry 0 – 2 mm	23.7 (19.8 – 25.9)	22.0 (20.6 – 24.8)	-1.3	-3.5 to 1.3	0.77
Total cornea densitometry 2 – 6 mm	19.0 (17.1 – 20.3)	18.0 (16.4 – 18.8)	-1.1	-2.6 to 0.2	0.77
Total cornea densitometry 6 – 10 mm	13.7 (13.4 – 15.4)	14.6 (13.7 – 16.1)	0.5	-0.5 to 1.5	0.77

CI – Confidence interval; *Mann–Whitney U test; †Hodges–Lehmann median difference. P values were adjusted using the Benjamini–Hochberg false discovery rate (FDR) correction.

Table S4. Differences in corneal densitometry between the standard and accelerated CXL protocols at nine months post-procedure.

9 months after CXL	Median (interquartile range)		†Difference	95% CI	*P**
	Standard protocol	Accelerated protocol			
Anterior (120 µm) cornea 0 – 2 mm	24.6 (21.4 – 29.7)	23.0 (20.6 – 24.8)	-2	-5.1 to 0.3	0.14
Anterior (120 µm) cornea 2 – 6 mm	18.9 (16.9 – 21.0)	18.6 (16.9 – 20.2)	-0.2	-2.2 to 1.3	0.78
Anterior (120 µm) cornea 6 – 10 mm	14.3 (13.6 – 16.4)	15.8 (14.6 – 17.1)	1.1	0.1 to 2.3	**0.06**
Center cornea layer 0 – 2 mm	19.1 (16.4 – 21.1)	18.4 (16.3 – 19.4)	-1.2	-3.1 to 0.5	0.32
Center cornea layer 2 – 6 mm	14.7 (13.5 – 15.8)	14.4 (13.5 – 15.5)	-0.3	-1.5 to 0.8	0.72
Center cornea layer 6 – 10 mm	11.9 (11.3 – 12.6)	13.0 (12.2 – 14.4)	1.2	0.4 to 2.1	**0.02**
Posterior (60 µm) cornea 0 – 2 mm	13.1 (12.6 – 14.9)	13.1 (12.2 – 14.5)	-0.2	-1.3 to 1.1	0.76
Posterior (60 µm) cornea 2 – 6 mm	11.3 810.9 – 12.2)	11.7 (10.9 – 12.5)	0.2	-0.5 to 0.9	0.64
Posterior (60 µm) cornea 6 – 10 mm	10.6 (10.1 – 11.4)	12.0 (11.5 – 13.4)	1.5	0.6 to 2.6	**0.006**
Total cornea densitometry 0 – 2 mm	19.2 (16.8 – 21.5)	17.8 (16.4 – 20.4)	-1.1	-3.0 to 0.9	0.81
Total cornea densitometry 2 – 6 mm	15.0 (13.8 – 16.3)	14.6 (13.9 – 16.2)	-0.1	-1.4 to 0.9	0.81
Total cornea densitometry 6 – 10 mm	12.3 (11.8 – 13.3)	13.6 (12.9 – 14.9)	1.3	0.3 to 2.2	**0.02**

CI – Confidence interval; *Mann–Whitney U test; †Hodges–Lehmann median difference. P values were adjusted using the Benjamini–Hochberg false discovery rate (FDR) correction. Bold denotes statistical significance after adjustment.

Table S5. Corneal densitometry changes across time points for the standard and accelerated CXL protocols.

	Median (interquartile range)				*P**
	Before CXL (1)	1 month after CXL (2)	3 months after CXL (3)	9 months after CXL (4)	
**Standard Dresden Protocol - 3mW**					
Anterior (120 µm) cornea 0 – 2 mm	21.2 (19.4 – 21.9)	30.3 (27.8 – 31.2)	29.8 (24.9 – 35.2)	24.6 (21.9 – 29.7)	**<0.001**†
Anterior (120 µm) cornea 2 – 6 mm	17.8 (17.4 – 18.7)	24.1 (22.3 – 26.6)	24.2 (20.0 – 27.4)	19.3 (17.1 – 21.1)	**<0.001**‡
Anterior (120 µm) cornea 6 – 10 mm	15.8 (15.4 – 16.8)	17.5 (16.8 – 18.6)	16.9 (15.9 – 18.2)	14.3 (13.7 – 16.3)	**<0.001**†
Center cornea layer 0 – 2 mm	15.5 (15.3 – 16.8)	23.1 (22.5 – 24.3)	24.3 (19.3 – 24.8)	19.1 (16.3 – 22.2)	**<0.001**§
Center cornea layer 2 – 6 mm	13.8 (13.3 – 14.5)	20.0 (19.3 – 23.0)	17.8 (16.7 – 19.8)	14.7 (13.4 – 15.9)	**<0.001**ǁ
Center cornea layer 6 – 10 mm	12.2 (11.9 – 13.3)	14.1 (12.8 – 14.5)	12.7 (12.5 – 14.0)	11.7 (11.3 – 12.4)	**<0.001**‡
Posterior (60 µm) cornea 0 – 2 mm	12.3 (11.8 – 13.0)	17.2 (15.1 – 19.9)	16.7 (14.6 – 17.1)	13.1 (12.2 – 14.9)	**<0.001**†
Posterior (60 µm) cornea 2 – 6 mm	11.4 (11.1 – 11.9)	16.3 (14.6 – 17.7)	13.7 (12.6 – 14.5)	11.3 (11.0 – 12.2)	**<0.001**ǁ
Posterior (60 µm) cornea 6 – 10 mm	11.1 (10.5 – 12.1)	11.8 (10.7 – 12.8)	11.4 (10.7 – 12.7)	10.3 (10.1 – 11.3)	**<0.001**‡
Total cornea densitometry 0 – 2 mm	16.3 (15.7 – 17.1)	23.0 (22.2 – 25.3)	23.7 (19.6 – 25.9)	19.2 (16.7 – 22.2)	**<0.001**†
Total cornea densitometry 2 – 6 mm	14.3 (13.9 – 15.0)	19.8 (18.8 – 22.2)	18.8 (16.6 – 20.3)	15.0 (13.9 – 16.4)	**<0.001**‡
Total cornea densitometry 6 – 10 mm	13.1 (12.7 – 13.9)	14.8 (13.5 – 15.2)	13.5 (13.1 – 14.8)	12.1 (11.7 – 13.2)	**<0.001**‡
**Accelerated protocol - 9mW**					
Anterior (120 µm) cornea 0 – 2 mm	21.0 (19.5 – 22.9)	27.9 (24.8 – 29.9)	28.4 (26.2 – 29.7)	22.3 (20.7 – 25.5)	**<0.001**‡

Anterior (120 µm) cornea 2 – 6 mm	18.3 (17.6 – 19.1)	23.4 (21.4 – 24.9)	22.9 (21.0 – 24.7)	18.3 (17.1 – 20.6)	**<0.001**[‡]
Anterior (120 µm) cornea 6 – 10 mm	17.2 (16.2 – 17.7)	18.3 (16.2 – 19.5)	17.1 (16.1 – 18.1)	15.8 (14.7 – 16.8)	**<0.001**[**]
Center cornea layer 0 – 2 mm	15.9 (15.5 – 17.0)	22.5 (19.2 – 28.3)	21.8 (20.4 – 24.3)	17.9 (16.3 – 20.4)	**<0.001**[†]
Center cornea layer 2 – 6 mm	14.1 (13.6 – 15.1)	18.9 (18.1 – 21.0)	17.4 (15.5 – 18.6)	14.7 (13.6 – 15.8)	**<0.001**[ǁ]
Center cornea layer 6 – 10 mm	13.9 (12.9 – 14.7)	14.5 (12.9 – 15.9)	13.6 (13.0 – 14.9)	13.0 (12.3 – 14.2)	**0.001**[**]
Posterior (60 µm) cornea 0 – 2 mm	12.6 (11.8 – 13.5)	17.7 (14.6 – 19.3)	15.3 (14.1 – 18.6)	13.5 (12.3 – 15.2)	**<0.001**[†]
Posterior (60 µm) cornea 2 – 6 mm	11.9 (11.5 – 12.6)	14.9 (13.6 – 15.7)	13.1 (12.1 – 13.9)	11.9 (11.3 – 12.6)	**<0.001**[ǁ]
Posterior (60 µm) cornea 6 – 10 mm	12.8 (11.9 – 13.2)	12.8 (11.5 – 14.7)	12.6 (11.8 – 13.7)	11.9 (11.5 – 13.2)	0.15
Total cornea densitometry 0 – 2 mm	16.6 (15.9 – 17.9)	22.5 (19.6 – 26.4)	22.0 (20.6 – 24.2)	17.7 (16.5 – 20.6)	**<0.001**[‡]
Total cornea densitometry 2 – 6 mm	14.9 (14.4 – 15.5)	18.9 (18.1 – 21.1)	18.0 (16.3 – 18.7)	14.9 (13.9 – 16.3)	**<0.001**[ǁ]
Total cornea densitometry 6 – 10 mm	14.6 (13.7 – 15.2)	15.3 (13.5 – 16.7)	14.3 (13.6 – 15.8)	13.6 (12.9 – 14.6)	**0.002**[**]

*Friedman test (post hoc Conover test); [†]at P < 0.05, significant differences between: (1) vs. (2, 3, 4); (4) vs. (2, 3); [‡]at P < 0.05, significant differences between all measurements; [§]at P < 0.05, significant differences between: (1) vs. (2, 3, 4); [ǁ]at P < 0.05, significant differences between: (1) vs. (2, 3); (2) vs. (3, 4); (3) vs. (4); **At P < 0.05, significant differences between: (1) vs. (2); (2, 3) vs. (4)

Table S6. ANCOVA results for corneal densitometry after CXL, adjusted for age and baseline densitometry

Time after CXL	Effect	F (1, df)	P value	Partial η²	F (1, df)	P value	Partial η²	F (1, df)	P value	Partial η²
		**Anterior (120 µm) cornea 0 – 2 mm**			**Anterior (120 µm) cornea 2 – 6 mm**			**Anterior (120 µm) cornea 6 – 10 mm**		
	3 mW vs. 9 mW	0	> 0,99	<0,001	0,424	0,52	0,014	0,012	0,91	0,000
1 month	Age	4,24	**0,04**	0,124	4,835	**0,04**	0,139	1,380	0,25	0,044
	Baseline	8,50	**0,007**	0,221	16,029	**<0,001**	0,348	46,18	**<0,001**	0,606
	3 mW vs. 9 mW	1,007	0,32	0,030	2,727	0,11	0,076	1,780	0,19	0,151
3 month	Age	0,282	0,59	0,009	0,011	0,92	0,000	0,014	0,91	0,000
	Baseline	2,958	0,09	0,082	4,727	**0,04**	0,125	44,38	**<0,001**	0,574
	3 mW vs. 9 mW	1,129	0,29	0,034	0,114	0,74	0,004	0,230	0,64	0,007
9 month	Age	0,011	0,92	0,000	0,011	0,92	0,000	1,668	0,21	0,050
	Baseline	1,926	0,18	0,057	1,754	0,20	0,052	17,65	**<0,001**	0,355
		**Center cornea layer 0 – 2 mm**			**Center cornea layer 2 – 6 mm**			**Center cornea layer 6 – 10 mm**		
	3 mW vs. 9 mW	0,908	0,34	0,029	0,002	0,96	0,000	0,000	0,99	0,000
1 month	Age	2,03	0,16	0,063	5,034	**0,03**	0,144	3,369	0,08	0,101
	Baseline	3,91	0,06	0,115	9,615	**0,004**	0,243	66,55	**<0,001**	0,689
	3 mW vs. 9 mW	0,159	0,69	0,005	2,960	0,09	0,082	1,185	0,28	0,035
3 month	Age	0,683	0,41	0,020	0,066	0,79	0,002	0,018	0,89	0,001
	Baseline	2,942	0,09	0,082	8,489	**0,006**	0,205	84,89	**<0,001**	0,72
	3 mW vs. 9 mW	1,108	0,30	0,033	0,251	0,62	0,008	0,327	0,57	0,010
9 month	Age	0,068	0,79	0,002	0,075	0,79	0,002	0,977	0,33	0,030
	Baseline	1,655	0,21	0,049	0,857	0,36	0,026	57,81	**<0,001**	0,644
		**Posterior (60 µm) cornea 0 – 2 mm**			**Posterior (60 µm) cornea 2 – 6 mm**			**Posterior (60 µm) cornea 6 – 10 mm**		
	3 mW vs. 9 mW	0,377	0,54	0,012	0,322	0,58	0,011	0,224	0,64	0,007
1 month	Age	2,853	0,10	0,087	6,476	**0,02**	0,178	4,286	0,05	0,125
	Baseline	14,06	**0,001**	0,319	11,75	**0,002**	0,282	73,49	**<0,001**	0,710
	3 mW vs. 9 mW	0,429	0,52	0,013	2,594	0,12	0,073	0,223	0,64	0,007
3 month	Age	0,009	0,93	0,000	0,019	0,89	0,001	0,739	0,40	0,022
	Baseline	4,525	**0,04**	0,121	9,394	**0,004**	0,222	59,54	**<0,001**	0,643
	3 mW vs. 9 mW	0,251	0,62	0,008	0,135	0,72	0,004	0,603	0,44	0,018
9 month	Age	0,400	0,53	0,012	0,126	0,73	0,004	0,793	0,38	0,024
	Baseline	0,426	0,52	0,013	0,029	0,87	0,001	54,36	**<0,001**	0,629
		**Total cornea densitometry 0 – 2 mm**			**Total cornea densitometry 2 – 6 mm**			**Total cornea densitometry 6 – 10 mm**		
1 month	3 mW vs. 9 mW	0,061	0,81	0,002	0,011	0,92	0,000	0,173	0,68	0,006

	Age	1,449	0,24	0,046	5,754	**0,02**	0,161	2,053	0,16	0,064
	Baseline	0,460	0,50	0,015	11,936	**0,002**	0,285	54,885	**<0,001**	0,647
3 month	3 mW vs. 9 mW	0,846	0,36	0,025	2,796	0,10	0,078	0,443	0,51	0,013
	Age	0,096	0,76	0,003	0,064	0,80	0,002	0,017	0,89	0,001
	Baseline	1,015	0,32	0,030	6,301	**0,02**	0,160	56,720	**<0,001**	0,632
9 month	3 mW vs. 9 mW	3,019	0,09	0,086	0,072	0,79	0,002	0,781	0,38	0,020
	Age	0,445	0,51	0,014	0,063	0,80	0,002	1,336	0,26	0,040
	Baseline	6,309	**0,02**	0,165	1,375	0,25	0,041	30,444	**<0,001**	0,488

Bold denotes statistical signigicance.

Table S7. Within-group changes in corneal densitometry before and nine months after CXL for the standard and accelerated protocols.

	**Median (interquartile range)**		†Difference	95% CI	*P**
	**Before CXL**	**9 month after CXL**			
**Standard protocol**					
Anterior (120 µm) cornea 0 – 2 mm	21.3 (19.5 – 23.2)	24.6 (21.4 – 29.7)	3.8	1.7 to 7.3	**0.01**
Anterior (120 µm) cornea 2 – 6 mm	17.8 (17.3 – 18.7)	18.9 (16.9 – 21.0)	0.5	-0.7 to 3.5	0.54
Anterior (120 µm) cornea 6 – 10 mm	15.8 (14.4 – 17.3)	14.3 (13.6 – 16.4)	-1.5	-2.2 to -0.6	**0.04**
Center cornea layer 0 – 2 mm	15.5 (15.4 – 16.8)	19.1 (16.4 – 21.1)	3.3	1.5 to 5.8	**0.006**
Center cornea layer 2 – 6 mm	13.8 (13.3 – 14.7)	14.7 (13.5 – 15.8)	0.5	-0.4 to 2.4	**0.02**
Center cornea layer 6 – 10 mm	12.4 (11.9 – 13.7)	11.9 (11.3 – 12.6)	-0.7	-1.2 to 0	0.05
Posterior (60 µm) cornea 0 – 2 mm	12.3 (11.8 – 13.3)	13.1 (12.6 – 14.9)	1.0	0.1 to 2.6	0.61
Posterior (60 µm) cornea 2 – 6 mm	11.4 (11.1 – 12.1)	11.3 810.9 – 12.2)	-0.2	-0.7 to 1.0	0.52
Posterior (60 µm) cornea 6 – 10 mm	11.1 (10.6 – 12.5)	10.6 (10.1 – 11.4)	-0.5	-0.9 to 0.2	0.09
Total cornea densitometry 0 – 2 mm	16.3 (15.7 – 17.2)	19.2 (16.8 – 21.5)	2.7	1.2 to 5.3	**0.009**
Total cornea densitometry 2 – 6 mm	14.3 (13.8 – 15.3)	15.0 (13.8 – 16.3)	0.2	-0.6 to 2.1	0.25
Total cornea densitometry 6 – 10 mm	13.2 (12.7 – 14.5)	12.3 (11.8 – 13.3)	-0.9	-1.4 to -0.2	0.05
**Accelerated protocol**					
Anterior (120 µm) cornea 0 – 2 mm	21.1 (20.1 – 23.4)	23.0 (20.6 – 24.8)	1.5	-1.2 to 4.1	0.32
Anterior (120 µm) cornea 2 – 6 mm	18.3 (17.8 – 19.7)	18.6 (16.9 – 20.2)	-0.03	-1.6 to 1.8	0.97
Anterior (120 µm) cornea 6 – 10 mm	17.4 (16.3 – 18.3)	15.8 (14.6 – 17.1)	-1.3	-2.1 to -0.6	**0.03**
Center cornea layer 0 – 2 mm	16.2 (15.6 – 17.1)	18.4 (16.3 – 19.4)	1.6	0.3 to 3.3	0.06
Center cornea layer 2 – 6 mm	14.2 (13.6 – 14.9)	14.4 (13.5 – 15.5)	0.2	-0.8 to 1.2	0.79
Center cornea layer 6 – 10 mm	14.0 (13.2 – 15.3)	13.0 (12.2 – 14.4)	-0.8	-1.3 to -0.2	**0.03**
Posterior (60 µm) cornea 0 – 2 mm	12.6 (11.9 -13.3)	13.1 (12.2 – 14.5)	0.9	-0.2 to 1.9	0.11
Posterior (60 µm) cornea 2 – 6 mm	11.9 (11.5 – 12.5)	11.7 (10.9 – 12.5)	-0.2	-0.8 to 0.4	0.39
Posterior (60 µm) cornea 6 – 10 mm	12.8 (12.3 – 13.7)	12.0 (11.5 – 13.4)	-0.6	-1.0 to -0.1	0.07
Total cornea densitometry 0 – 2 mm	16.6 (16.1 – 17.9)	17.8 (16.4 – 20.4)	0.8	-1.0 to 2.3	0.40
Total cornea densitometry 2 – 6 mm	15.0 (14.5 – 15.5)	14.6 (13.9 – 16.2)	-0.1	-1.0 to 0.9	0.89
Total cornea densitometry 6 – 10 mm	14.7 (14.1 – 15.9)	13.6 (12.9 – 14.9)	-0.9	-1.5 to -0.3	**0.03**

CI – Confidence interval; *Wilcoxon test; †Hodges–Lehmann median difference. P values were adjusted using the Benjamini–Hochberg false discovery rate (FDR) correction. Bold denotes statistical significance after adjustment.
